# Pattern of physical activity can influence its efficacy on muscle and bone health in middle-aged men and women

**DOI:** 10.1007/s11332-018-0448-z

**Published:** 2018-03-29

**Authors:** Jin Luo, Alastair Ratcliffe, Jaswinder Chahal, Richard Brennan, Raymond Lee

**Affiliations:** 1 Department of Life Sciences, University of Roehampton, London, UK; 2 Faculty of Technology, University of Portsmouth, Portsmouth, UK; 3 School of Applied Sciences, London South Bank University, 103 Borough Road, London, SE1 0AA UK

**Keywords:** Aging, Accelerometry, Musculoskeletal health, Exercise prescription

## Abstract

**Purpose:**

This study aimed at investigating whether association between physical activity, and bone density and muscle strength depends on daily activity pattern.

**Methods:**

Loading dose of moderate-to-vigorous physical activity (MVPA) was measured using accelerometer on 54 men (*M*_age_ = 54.1 years) and 59 women (*M*_age_ = 52.1 years). Pattern of MVPA was quantified as number and length of MVPA bouts, and the length of break bouts between MVPA bouts. Knee extension torque (KET) and broadband ultrasound attenuation (BUA) of the calcaneus were also measured. Regression analysis was employed to examine the moderation effect of MVPA pattern.

**Results:**

Loading dose had a larger effect on BUA (*b* = .002, *p* = .035) and KET (*b* = .004, *p* = .01) with the increase of median length of MVPA bout, but had a smaller effect on KET with the increase of maximal length of break bout (*b* = − .015, *p* = .024).

**Conclusions:**

This study suggests that pattern of physical activity can influence its efficacy on muscle and bone health.

## Introduction

Middle age is associated with the deterioration in structure and function of musculoskeletal system [1, 2]. The gradual loss of mass and strength of bone and muscle during this period may lead to the development of diseases such as osteoporosis and sarcopenia in later life. Factors that contribute to this ageing-related decline include hormones [3], nutrition [4], and physical inactivity.

Physical activity is able to prevent or attenuate the loss of bone and muscle in the middle-aged men [2] and women [5]. To develop effective exercise interventions, it is important to understand the dose–response relationship between mechanical loading of physical activity and musculoskeletal health. It was found that loading dose of physical activity was associated with bone density and muscle strength in the middle-aged women [6]. However, this association only existed when loading intensity reached above moderate-to-vigorous level. Similar findings were also reported by other researchers showing that only physical activity with acceleration above moderate level was positively associated with hip bone mineral density (BMD) [7, 8], total body lean mass [9], and lower limb muscle strength [10, 11]. All these studies seem to suggest that moderate-to-vigorous physical activity (MVPA) is crucial for the adaptation of musculoskeletal system.

In recent years, accelerometers have been extensively used to assess physical activity. One major advantage of this method is its ability to objectively measure the dose of physical activity using various parameters, such as activity counts [12], time spent at different intensities of physical activities [13, 14], impact score [15], and loading dose [6]. Although these parameters can assess the total amount of moderate-to-vigorous physical activity during a day, one limitation is that they cannot provide information on the pattern of moderate-to-vigorous physical activity, for example, how MVPA bouts are distributed across a day, the length of time of each MVPA bout and the break between MVPA bouts. MVPA pattern might have significant influence on musculoskeletal adaptation to mechanical loading. Previous studies on animals have found that the same amount of mechanical loading might be able to induce different osteogenic response if the loading was distributed in different pattern (e.g. different bout length, different resting period between loading bouts) during a day [16]. However, there has been a lack of study to date which examined the influence of MVPA pattern on the dose–response relationship between mechanical loading and musculoskeletal health in older people. It is thus important to answer this research question for the development of optimal exercise regimens.

The aim of the current study was to investigate whether the association between loading dose, and bone density and muscle strength depends on patterns of MVPA in the middle-aged men and women.

## Methods

### Participants

Fifty-four men (*M*_age_ = 54.1 years; SD = 8.9) and 59 women (*M*_age_ = 52.1 years; SD = 7.6) were recruited. They were all recreationally active. The body mass index was 25.9 kg/m^2^ (SD = 3.3) for males, and 24.0 kg/m^2^ (SD = 3.6) for females. Participants were included in the study if they were free of musculoskeletal injury or disability, did not smoke, and physically fit and able enough to partake in the study. The study was approved under the procedures of the local Ethics Committee. All participants gave written informed consent before participating in the study.

### Sample size

Power calculation was conducted to determine the sample size for this study. G*Power software (version 3.1.9.2) was used [17], with the total number of predictors being set at 6 (age, gender, BMI, loading dose, pattern of MVPA, and the interaction between loading dose and pattern of MVPA). Based on the assumption that interaction between pattern of MVPA and loading dose would induce a medium-sized *R*^2^ increase (Δ*R*^2^ = .10), the power calculation showed that a sample size of 100 was required to achieve a power of .9 at alpha level of .05.

### Measurements

#### Physical activity

A miniature accelerometer (size 39 × 23 × 72 mm; weight 16 g, model 145B, MSR Electronics GmbH, Switzerland) was attached to the lower back of the participants, and programmed to record 10 h (9 a.m. to 7 p.m.) of three-axis acceleration data at a sampling rate of 20 Hz. The accelerometer was attached using double-sided medical tape onto the skin over the sacrum. Participants were instructed not to deviate from normal activities. The accelerometer was returned after the 10-h testing period for data collection.

#### Bone density

A bone ultrasound scanner (McCue Cuba Clinical Machine Version 2.6, Hampshire, England) was used to measure broadband ultrasound attenuation (BUA) of the calcaneus on the right foot.

#### Muscle strength

Dynamic knee extension torque (KET) was measured on the right leg using an isokinetic dynamometer (Cybex Norm, Computer Sports Medicine Inc., Stoughton, MA, USA). Each participant was seated in a chair fixed at 85° recline angle. Straps were fastened at the chest, thigh and ankle to ensure support whilst extending the knee with force. The centre of rotation of the dynamometer lever arm was aligned with the lateral condyle of the right tibia of the participant. Range of motion was tested and secured against safety locks. KET was tested at a set angular velocity of 60 deg/s. Peak torque was collected from a set of five repetitions, with verbal encouragement offered throughout to ensure maximum effort. A brief warm-up on the treadmill preceded a familiarisation set of five repetitions.

### Data analysis

The raw accelerometer data were processed by a customized MATLAB program (v.7.10.0, R2013a; the Mathworks, Inc, Natick, Massachusetts, USA) which calculated the resultant acceleration and filtered the data using a Butterworth band pass filter (.1–6 Hz) to remove static gravitational acceleration and noise [18].

The 10-h acceleration data were then split into 7200 consecutive segments, each 5 s long. Fast Fourier transformation was used to obtain Fourier series of each segment. Loading intensity was then calculated for each segment as [18]

$$ {\text{LI}} = \mathop \sum \limits_{{{\text{fi}} = 0.1}}^{{6{\text{Hz}}}} \left( {{\text{Ai}} \times {\text{fi}}} \right)/g $$

where LI is loading intensity normalized to body weight (BW/s), Ai is acceleration (m/s^2^) at frequency fi, and *g* is gravitational acceleration (9.81 m/s^2^).

Based on its loading intensity value each segment was categorised into one of the three categories—very light (LI < 5 BW/s), light (5 BW/s < LI < 10 BW/s), moderate-to-vigorous (LI > 10 BW/s) [6]. Previous study [18] showed that typical activities associated with these categories were: very light—slow walking, normal walking, and ascending and descending stairs; light—fast walking; moderate-to-vigorous—slow to fast running. Loading dose of physical activity was then calculated at each intensity category as [6]

$$ {\text{LD}} = \ln \left( {1 + \mathop \sum \limits_{k} 5 \times {\text{LI}}} \right) $$

where LD is loading dose, *k* is the number of segments in a specific intensity category.

**Fig. 1:**
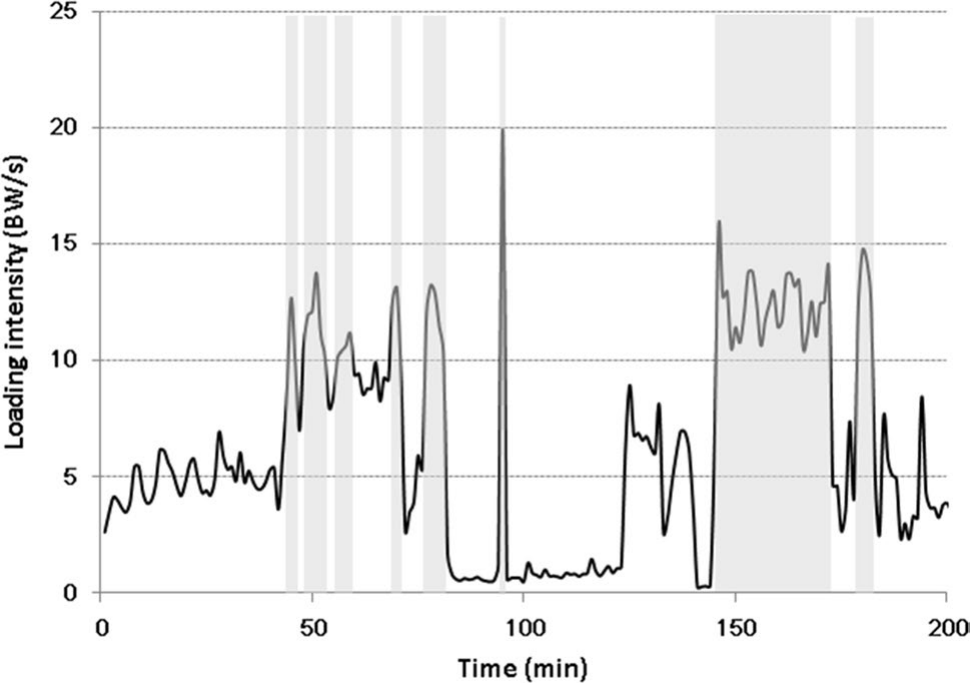
A section of loading intensity curve from one participant. Each grey bar represents a MVPA bout. The white bar between two neighboring grey bars represents a break bout

A MVPA bout was defined as consecutive 5-s segments (without break) that had loading intensity higher than 10 BW/s. A break bout was defined as the segment(s) between two consecutive MVPA bouts (Fig. 1). Pattern of MVPA bouts were examined using following parameters: number of MVPA bouts (No_MVPA), defined as the total number of MVPA bouts during the 10-h recording period; Median length of MVPA bout (ML_MVPA), defined as median length of all MVPA bouts during the 10-h recording period; Maximal length of MVPA bout (MaxL_MVPA), defined as maximal length of all MVPA bouts during the 10-h recording period; Median length of break bout (ML_break), defined as median length of all break bouts during the 10-h recording period; Maximal length of break bout (MaxL_break), defined as maximal length of all break bouts during the 10-h recording period.

### Statistics

Association between loading dose, and BUA and KET was first examined using multiple linear regression models (model 1–3), with loading dose at very light intensity (LD_VLPA), light intensity (LD_LPA), or moderate-to-vigorous intensity (LD_MVPA) being entered individually as the independent variable. Moderation analysis [19, 20] was then conducted by entering each parameter for pattern of MVPA (i.e. number of MVPA bouts, median length of MVPA bout, maximal length of MVPA bout, median length of break bout, or maximal length of break bout) individually into model 3 as the moderation variable. As loading dose was normalized to body weight, BUA and KET were also normalized to body weight before being entered into regression analysis.

All multiple linear regression models were adjusted for age, gender, and BMI. Multi-collinearity between independent variables was checked by variance inflation test (VIF). Regression coefficient (*b*) and its 95% confidence interval (95% CI) were presented for potential associations. All statistical analyses were performed with SPSS 22.0 (IBM, Armonk, NY, USA) with the PROCESS command tool for moderation analysis [19, 20]. For all analyses, *p* values less than .05 were considered to be significant.

## Results

As seen from Table 1 there were less than 20 MVPA bouts during the 10-h recording period in 75 percent of participants. The length of MVPA bouts tended to be very short: The 75th percentile of median length of MVPA bouts was less than 10 s, and the 75th percentile of maximal length of MVPA bouts was less than 30 s. These few and short MVPA bouts were separated by long break bouts, with more than 50% of participants’ median break length longer than an hour, and more than 90% of participants’ maximal break length longer than 2 h.

**Table 1:** Loading dose and pattern of MVPA in female and male participants (*N* = 113)

Percentile	10th	25th	50th	75th	90th
LD_VLPA	9.71	9.88	10.09	10.29	10.42
LD_LPA	5.60	6.77	7.65	8.53	9.04
LD_MVPA	0	4.05	5.71	7.60	9.68
No_MVPA	0	1	3	13	28
ML_MVPA (s)	0	5	5	5	10
MaxL_MVPA (s)	5	5	5	20	186
ML_break (h)	.01	.15	1.12	4.99	9.99
MaxL_break (h)	2.76	4.13	6.54	9.36	9.99

*LD_VLPA* loading dose at very light intensity, *LD_LPA* loading dose at light intensity, *LD_MVPA* loading dose at moderate-to-vigorous intensity, *No_MVPA* number of MVPA bout, *ML_MVPA* median length of MVPA bouts, *MaxL_MVPA* maximal length of MVPA bout, *ML_break* median length of break bout, *MaxL_break* maximal length of break bout

Loading dose at moderate-to-vigorous intensity were positively associated with BUA (standardized regression coefficient b* = .314, *p* < .001 for model 3) and KET (b* = .190, *p* = .023 for model 3). In contrast, loading dose at very light or light intensity had no significant association with BUA (b* = .019, *p* = .835 for model 1; b* = .071, *p* = .429 for model 2) or KET (b* = − .019, *p* = .816 for model 1; b* = .092, *p* = .272 for model 2) (Table 2). In model 3 for BUA the standardized regression coefficient for age was b*_age_ = − .167 (*p* = .049), while in model 3 for KET the standardized regression coefficient for age was b*_age_ = − .426 (*p* < .001). These results indicated that loading dose at moderate-to-vigorous level had comparable effect sizes as age in the multiple linear regression model (model 3).

**Table 2:** Loading dose as independent predictor of bone density and muscle strength (*N* = 113)

	Model	BUA		KET	
		*R* ^2^	*b* [95% CI]	*R* ^2^	*b* [95% CI]
LD_VLPA	1	.168	.018 [− .152, .187]	.315	− .035 [− .333, .263]
LD_LPA	2	.173	.019 [− .022, .052]	.323	.036 [− .029, .101]
LD_MVPA	3	.262	.026 [.012, .040]***	.349	.029 [.004, .055]*

Linear regression model adjusted for age, gender, and BMI

*LD_VLPA* loading dose at very light intensity, *LD_LPA* loading dose at light intensity, *LD_MVPA* loading dose at moderate-to-vigorous intensity

* *p* < .05; ** *p* < .01; *** *p* < .001

The effect of loading dose at moderate-to-vigorous intensity on BUA or KET was moderated by median length of MVPA bout (*b* = .002, *p* = .035 for BUA and *b* = .004, *p* = .01 for KET) (Table 3). With the increase of median length of MVPA bout, loading dose had a larger effect on BUA and KET. For example, regression coefficient for association between loading dose and BUA increased from *b* = .025 (*p* = .001) at 10th percentile of ML_MVPA to *b* = .042 (*p* < .001) at 90th percentile of ML_MVPA. Similarly, regression coefficient for association between loading dose and KET also increased from *b* = .038 (*p* = .004) at 10th percentile of ML_MVPA to b = .083 (*p* < .001) at 90th percentile of ML_MVPA.

**Table 3:** Moderation effect of MVPA pattern on association between loading dose, and BUA and KET (*N* = 113)

	BUA		KET	
	*R* ^2^	*b* [95% CI]	*R* ^2^	*b* [95% CI]
LD_MVPA No_MVPA LD_MVPA*No_MVPA	.267	.038 [.002, .061]* − .002 [− .011, .007] .000 [− .002, .002]	.389	.024 [− .026, .074] .001 [− .018, .020] .001 [− .003, .006]
LD_MVPA ML_MVPA LD_MVPA*ML_MVPA	.285	.049 [.022, .075]*** − .008 [− .016, − .000]* .002 [.000, .003]*	.399	.102 [.046, .158]*** − .024 [− .041, − .007]** .004 [.001, .008]*
LD_MVPA MaxL_MVPA LD_MVPA*MaxL_MVPA	.280	.007 [− .034, .049] .001 [− .001, .003] − .001 [− .001, .000]	.352	.009 [− .072, .091] .001 [− .003, .005] − .000 [− .001, .001]
LD_MVPA ML_break LD_MVPA*ML_break	.267	.029 [− .009, .069] − .001 [− .044, .041] − .002 [− .008, .004]	.385	.039 [− .041, .119] − .015 [− .099, .068] − .010 [− .021, .000]
LD_MVPA MaxL_break LD_MVPA*MaxL_break	.263	.030 [.003, .057]* .003 [− .026, .033] − .002 [ .009, .006]	.389	.045 [.002, .087]* − .003 [− .051, .045] − .015 [− .027, − .002]*

Linear regression model adjusted for age, gender, and BMI

*LD_MVPA* loading dose at moderate-to-vigorous intensity, *No_MVPA* number of MVPA bout, *ML_MVPA* median length of MVPA bout, *MaxL_MVPA* maximal length of MVPA bout, *ML_break* median length of break bout, *MaxL_break* maximal length of break bout

* *p* < .05; ** *p* < .01; *** *p* < .001

The effect of loading dose at moderate-to-vigorous intensity on KET was moderated by maximal length of break bout (*b* = − .015, *p* = .024) (Table 3). When maximal length of break bout was long, for example, at 90th percentile level, there was no significant association between loading dose and KET (*b* = − .002, *p* = .938). However, the association between loading dose and KET became significant with the decrease of maximal length of break bout, for example, the association was significant at 50th percentile (*b* = .042, *p* = .046), 25th percentile (*b* = .079, *p* = .015), and 10th percentile (*b* = .100, *p* = .013) of maximal length of break bout.

## Discussion

The current study found that MVPA in the middle-aged was in the form of very short bouts distributed across the day. Loading dose of MVPA was associated with muscle strength and bone density, with an effect size comparable to age. However, the efficacy of MVPA loading dose depends on its daily pattern: It became larger with the increase of median MVPA bout length and the decrease of maximal break bout length.

A main strength of our study is that mechanical loading of physical activity was objectively assessed in natural environment using accelerometer. The size of the accelerometer used was very small so that measurement could be done with little interference to participants’ normal daily activity. The method of assessing loading dose considered loading magnitude and loading rate (frequency) in its calculation [6, 18]. This is likely to provide a more accurate measurement of bone loading as both loading magnitude and loading frequency are important parameters that determine bone adaptation [21, 22].

The current study quantitatively examined the pattern of MVPA in daily activity. It was found that MVPA was in the form of very short bouts distributed across the day, with most of its bout length less than 10 s long (Table 1). These short MVPA bouts are separated by long period of breaks (usually longer than an hour) where loading intensity were lower than moderate level. As a result, the number of MVPA bouts during a day is quite low in the middle-aged, with most participants having only less than 20 MVPA bouts during the whole 10-h recording period. Our results are in line with a previous study [6] which found that the 50th and 75th percentile of the duration of moderate-to-vigorous activity during a day is 7.5 and 57 s, respectively, for middle-aged women. Other studies also found that MVPA was only a very small part of the total activity during a day. Chastin et al. [13] found that percentages of MVPA of a day for 2117 men and women between age 50 and 59 were 2.9 and 1.7%, respectively. This further decreased to 2.1 and 1.3% for men and women, respectively, for age between 60 and 69. It was also found that the number of high impact counts (with acceleration > 3 g) was around 30 in adolescents per day [9], but decreased to less than 8 per week for the elderly [23]. These findings suggest that MVPA is rare during daily activity, and the amount of MVPA decreases with ageing. Although MVPA is rare during daily activity, it is important for musculoskeletal health. As shown in our results, loading doses at moderate and vigorous intensity were associated with BUA and KET, while loading dose in very light or light intensity was not. This threshold effect on association between physical activity and musculoskeletal adaptation has been reported in several previous studies [7–9, 24].

For the first time, the current study investigated the moderation effect of patterns of MVPA on the association between loading dose, and bone density and muscle strength in older people. Our findings are in line with previous experiments investigating the biological response of bone to mechanical stimuli. It was found on a functionally isolated avian bone that four loading cycles per bout each day over 6 weeks could not induce any bone remodeling, but increase of loading cycles to 36 per bout could induce bone adaptive response [21] suggesting that the number of loading cycles in a bout needs to be over a certain threshold to induce osteogenic effect. This can explain our finding that the increase of median length of MVPA bouts can improve its efficacy on bone density. As shown in Table 1, the median length of MVPA bouts in half of the participants is only 5 s long. This means that a large portion of MVPA bouts during a day did not reach the length threshold for osteogenic effect. On the other hand, the increase of median length of MVPA bouts can ensure that there are more MVPA bouts with its length above the threshold to improve the efficacy of MVPA loading dose. The current study also found that median length of MVPA bout had positive moderation effect on KET. This is consistent with muscle physiology that multiple repetitions of muscle contraction are needed during an exercise bout to stimulate muscle protein synthesis [25].

The current study also found that maximal length of break bout had a negative moderation effect on the efficacy of MVPA. With the increase of maximal length of break bout, there was a loss of association between loading dose and KET. This result may be related to the deteriorating effect of sedentary behavior on muscle, which can lead to an increased risk of sarcopenia [26]. However, it should be pointed out that the current study had not specifically quantified sedentary time as the length of break bout included all physical activities below moderate intensity level.

The findings from this study have several clinical implications. We found that loading dose of MVPA had a comparable effect size as age in our multiple regression analysis. This suggests that mechanical loading from MVPA can play an important role in the protection against ageing-related diseases such as osteoporosis and sarcopenia. Our results also suggest that the effectiveness of mechanical loading is dependent on MVPA pattern. It is thus important to consider this factor in the future when studying the dose–response relationship between physical activity and musculoskeletal health.

The main limitation of the current study is its cross-sectional design. No causal relationship can be inferred from our results. Another limitation is that bone density was only measured on heel bone, and muscle strength was only measured on knee extensor in this study. Future studies should further investigate the moderation effect of MVPA pattern on the association between loading dose, and bone density and muscle strength in different body locations.

## Conclusions

In conclusion, the results of the present study suggest that moderate-to-vigorous physical activity plays an important role in the protection against ageing-related diseases such as osteoporosis and sarcopenia. However, the efficacy of MVPA depends on its daily pattern: it becomes larger with the increase of median length of MVPA bout and the decrease of maximal length of break bout. Thus, pattern of moderate-to-vigorous physical activity is an important factor that should be considered in future studies on physical activity and musculoskeletal health.

## Abbreviations

BMD: Bone mineral density
MVPA: Moderate-to-vigorous physical activity
BMI: Body mass index
BUA: Broadband ultrasound attenuation
KET: Knee extension torque
BW: Body weight
LI: Loading intensity
LD: Loading dose
LD_VLPA: Loading dose of very light physical activity
LD_LPA: Loading dose of light physical activity
LD_MVPA: Loading dose of moderate-to-vigorous physical activity
No_MVPA: Number of moderate-to-vigorous physical activity bouts
ML_MVPA: Median length of moderate-to-vigorous physical activity bouts
MaxL_MVPA: Maximal length of moderate-to-vigorous physical activity bouts
ML_break: Median length of break bout
MaxL_break: Maximal length of break bout
